# Health of London during 1856

**Published:** 1857-10

**Authors:** John Webster


### ON THE HEALTH OF LONDON DURING 1856.

By JOHN WEBSTER, M.D., F.R.S., etc.

THE metropolitan population generally enjoyed, in 1856, like other portions of the British empire, a very satisfactory degree of public health; the total deaths being less numerous than during 1853-54 and 1855, notwithstanding the great increase of population. In 1856, the aggregate mortality, throughout all the metropolitan districts, numbered 56,786, or 4,720 fewer than during 1855; although that year was considered likewise salubrious. In reference to particular quarters of the year, a somewhat striking uniformity prevailed. The autumn, however, showed the smallest mortality; the deaths being in that quarter 14,066. In the previous three months—ending June 28th—14,069 were recorded. Next in amount comes the fourth quarter, when 14,118 fatal cases were reported; while the season which exhibited the largest death-rate were the months of January, February, and March, when 14,533 persons died. Taken altogether, every season appears therefore to have been uniformly salubrious; and if the average be calculated according to the actual inhabitants living in London, the ratio then ranged as only one death in every forty-six residents.

Such results constitute a very low mortuary scale; and, if compared with previous years, or with many other large capitals, either in Europe or elsewhere, it becomes even more remarkable. For instance, in Paris, the proportion reached one death in about 30 residents; and in New York one person died in at least every 25. Other towns—teeming with human beings, and similarly confined in very limited spaces—might be also now mentioned; but it seems superfluous, as all concurrent facts prove that London is by far the most healthy capital of the world, notwithstanding its enormous population, and the numerous existing causes of disease which still prevail.

I. *Diseases showing an increased Mortality.* Notwithstanding the marked salubrity of last year, several maladies manifested an augmented severity; and hence were followed by a larger number of deaths than during the twelve months immediately preceding. Thus, measles proved fatal to 1,445 persons against 864 in 1855. By diarrhœa, 2,251 died, instead of 2,061 in the same period; by dysentery, 165 against 142; by typhus, 2,645 in place of 2,332; by syphilis, 209 against 168; by nephria, 237 against 200; by diabetes, 50 against 38; and by carbuncle, 78 against 53; this, however, is not so great a mortality from that affection as occurred in 1854, when 91 fatal cases of carbuncle were recorded.

Although the total deaths by accidents, viewed in the aggregate, were rather fewer during 1856 than the previous year, the numbers being 1831, instead of 1803 reported during 1855; still, under several specific heads, there appears an augmentation. For example, 97 persons died by poison, instead of 85, in 1855; by suffocation, 174 instead of 158; by drowning, 378 instead of 324; and by wounds 112, instead of 100 in the former period. On the other hand, fewer persons lost their lives by fractures; the comparative numbers being 620 cases in 1856, instead of 652 during the previous year. These varied facts, regarding fatal casualties in London, are very interesting, in so far that they indicate that, however numerous may be the specific causes of violent deaths, throughout the immense metropolitan population, the average amount of such events, happening one year with another, seems to exhibit very little variation. For illustration, by drowning, during the past five years, viz., 1852-6, the total deaths were 353, 355, 344, 324, and 378 respectively. In fact, nearly the same number of residents have their mortal career thereby cut short annually in London, as also through other violent means, however different various collateral circumstances may often appear.

II. *Diseases proving less Fatal.* Several maladies have, on the other hand, exhibited a diminished rate of mortality, which sufficiently account for the late satisfactory condition of public health. This will be seen by the subjoined table.

| Diseases. | Deaths in 1855. | Deaths in 1856. |
|---|---|---|
| Small-pox | 1024 | 522 |
| Scarlet fever | 2602 | 1795 |
| Hooping cough | 2414 | 2078 |
| Influenza | 168 | 52 |
| Remittent fever | 104 | 88 |
| Erysipelas | 402 | 388 |
| Dropsy | 829 | 728 |
| Hydrocephalus | 1531 | 1351 |
| Phthisis | 7545 | 7196 |
| Cephalitis | 603 | 510 |
| Apoplexy | 1382 | 1306 |
| Paralysis | 1180 | 1133 |
| Insanity | 133 | 78 |
| Convulsions | 1937 | 1872 |
| Bronchitis | 5512 | 4482 |
| Pneumonia | 3992 | 3621 |
| Asthma | 728 | 585 |
| Enteritis, gastritis, and peritonitis | 656 | 550 |
| Hernia | 166 | 137 |
| Ovarian dropsy | 49 | 38 |
| Childbirth | 272 | 246 |
| Intemperance | 72 | 66 |
| Starvation | 35 | 28 |

III. *Diseases having the same Ratio of Deaths.* Analogous to results observed during preceding years, and notwithstanding the enormous yet ever changing population of the metropolis, various maladies often show an extraordinary uniformity in the number of deaths thereby produced.

| Diseases. | Deaths in 1855. | | Deaths in 1856. |
|---|---|---|---|
| Purpura | 51 | .... | 50 |
| Hæmorrhage | 213 | .... | 210 |
| Fistula | 19 | .... | 19 |
| Gout | 59 | .... | 60 |
| Laryngitis | 283 | .... | 283 |
| Ulceration of intestines | 122 | .... | 122 |
| Ileus | 150 | .... | 151 |
| Hepatitis | 181 | .... | 177 |
| Rheumatism | 233 | .... | 232 |

The above statements are exceedingly interesting, and well deserve the attention of medical statists, as likewise of those occupied in the investigation of contingencies affecting human life, and its probable duration.

The large amount of infants who fall a sacrifice to the "want of breast milk" constitutes a great calamity; and if it cannot be checked, the real source deserves unmeasured condemnation. Since this frequent cause of death has been noticed in the metropolis, it seems to have gone on every year augmenting. Thus, during 1848, the number of infants who died thereby was 171; in 1852, 276; and in 1856, 366, or double those recorded eight years before. Such facts ought to make an impression upon those—often amongst the highest ranks—who patronize a system which leads to such disastrous results. Irrespective of the very injurious consequences thus inflicted upon many innocent babes of the poor, nothing proves so beneficial both to the offspring as well as to the individual health of the parents as to follow the true dictates of nature, which say, "*Every mother should nurse her own child.*" Were this done, the above sad results would very rarely supervene. It has now become high time for the medical profession to interfere, and by every available means in their power endeavour to check the practice to which reference is here made. Exceptional cases may certainly sometimes occur, where the mother cannot suckle her own child; but then, greater care should always be taken by such persons, that the wet nurse's infant be not itself sacrificed.

Although an improved state of health, as a whole, prevailed generally throughout London during all last year, the mortality varied in particular districts. Thus, the ratio ranged low in the western and northern, contrasted with the eastern, portions of the metropolis. For example, Westminster, formerly considered less salubrious than the more elevated parts of western London, exhibited a decided decrease in the total number of deaths; which result has been ascribed, and justly, to measures of sanitary improvement recently effected. Again, in both Kensington and Islington — heretofore esteemed desirable neighbourhoods for residences—the mortality shows a decided increase.

The discrepancy manifested in reference to typhus, and its fatality, being a good criterion of the average health enjoyed by residents in particular localities, it becomes instructive to state, regarding this disease, that in the east districts—having a population of 485,522 persons—the deaths by typhus were 793, or 1 in 612; whereas, in the west districts—containing 376,427 inhabitants—the fatal cases were 326 by the same disease, or 1 in 1,124. Scarlatina also proved more than twice as fatal in the eastern as in the more western portions of London. These facts are in accordance with previous and general experience; the west and north districts of the metropolis being usually most salubrious, and hence far less affected by epidemics under ordinary circumstances.

Amongst the total 56,786 deaths recorded during last year, 10,381, or nearly one-fifth, occurred in charitable institutions, more than half being among inmates of workhouses, the majority of the latter category being females. It is worth noticing that, both in workhouses, and in the metropolitan general hospitals, the total mortality has, of late years, been regularly diminishing. Similar data, with regard to mortality, have lately been likewise obtained in military and naval hospitals, as also in lunatic asylums.

In prisons, although their actual population always averages upwards of 6000 persons, 81 deaths only were reported; 64 being males, and 17 females. This result shows the present marked salubrity of these establishments, altogether very different from what prevailed in and previously to the days of Howard, when to be committed to durance vile in any London jail was almost consigning the unfortunate individual to certain prospective death. Now, in many instances, confinement in prison becomes an efficacious means towards the restoration of physical health; whilst it at the same time often promotes moral improvement.

In the four metropolitan Lying-in-Hospitals, 14 deaths only occurred among all the puerperal women under treatment, during 1856. At the Queen Charlotte establishment, not one fatal case through child-birth was recorded. With respect to institutions for special diseases, it may be observed that the Fever and Consumption Hospitals alone seem worthy of remark on the present occasion; 279 deaths having occurred at the former, and 181 in the latter. At the Fever Hospital, the deaths were almost equal in both sexes, being 140 to 139; while by phthisis 114 males and 67 females died, or nearly two of the former to one of the latter. This statement, however, only shows that more male patients were then admitted, but it does not prove consumption to be in a higher degree fatal among them than among the other sex.

In the garrison of London, comprising the three regiments of guards, only 44 fatal cases by disease are reported during 1856, which makes a very small mortality.

*Increase of Population.* More births took place during last year than in any previous one; the total number of children then born being 86,833, exceeding by 30,047 the whole mortality recorded. Irrespective of the annual immigration constantly in operation, it is thus easy to explain the great increase of London during recent times, since more persons are born in every year within its precincts than those who die. Should events proceed at the same ratio as now, in a very short period London will then contain upwards of three millions of inhabitants, or more residents than many European kingdoms possess.

A few general observations may be now made in reference to one or two classes of persons; such remarks being, however, confined to parties chiefly occupied in mental occupations. Respecting the three learned professions, it appears that last year, amongst lawyers, one died in every 64 then living; amongst medical practitioners, one in 55; and amongst clergymen and religious teachers, one in every 37. Amongst law-students the proportion of deaths was much less than that exhibited by medical pupils; the rate of mortality in the former being only one in every 141, while in the latter it appears to have amounted to one death in every 62. In fact, these data would warrant the inference that the contingent probability of death was not only greater among medical men than among lawyers in active practice, but likewise when studying their respective professions.

Metropolitan physicians frequently attain very advanced ages. Thus, among the 68 Presidents of the College of Physicians, since its foundation in 1518, although the ages of only one-third of that number are known, eighteen had passed 70; ten of them were octogenarians and beyond, two of these being nonagenarians, the oldest having reached 93 at death; viz., Dr. Collins, who presided in 1695, and died in 1710.

At present, two members are alive who exceed 90 years, one being 92; while very recently three others died who had attained almost equal patriarchal ages, one being 93; thus showing that London physicians are often long lived, which fact becomes more remarkable when it is known that the class is not numerous. The proportion of deaths among merchants to the living ranged at one in 51 during 1856, but in commercial clerks it was one in 49; whereas, among commercial travellers, who are engaged in nearly similar pursuits, but comparatively lead a much more active life, and pass a great deal of their time in the open air, the rate appears as one in 64; thus showing the occupation of commercial travellers to be more salubrious than that of individuals confined in counting-houses and by sedentary employments.

While the deaths amongst schoolmasters reached so high as one in every 36, it was only one in every 72 music masters; that is, two teachers of youth in schools died against one who followed the more varied, less sedentary, and certainly much more agreeable duties of music-teaching. Of actors, lately, one in every 52 seems to have died; whereas, amongst musical performers, whose occupation is devoid of locomotion and much bodily exertion, the proportion of deaths was double, or less than one in every 25. The rate of mortality amongst annuitants ranged so low as only one in every 314, warranting the general inference of annuitants being long-livers, and that one of the best prescriptions to prolong human existence is the regular receipt of a cheque upon some bank, which will be always honoured.

Although the classes of persons to whom I am now about to refer are not strictly comprised amongst those engaged in mental occupations, still, as they do not live by bodily labour, and are otherwise interesting, while in several respects there prevails some resemblance in their respective conditions, a few remarks, illustrating the mortality met with in Chelsea and Greenwich pensioners, as also amongst the metropolitan police, will not be out of place. The two former are all old men, while the latter body is entirely composed of persons usually in the vigour of manhood. As might be expected, the mortality which residents of Greenwich Hospital reached was nearly one in twelve; whereas, at Chelsea, the ratio amongst old soldiers ranged at almost one in sixteen, or a third less than in veteran seamen, who are neither so healthy, nor generally attain such advanced ages as Chelsea pensioners. Throughout the metropolitan police, on the other hand, the deaths were reported at one in every 90.

This result is, however, not more singular than might be anticipated, considering the ages of policemen, and seeing they do not remain attached to their corps during their whole lifetime, as in the two former bodies of men; many of these having been also in foreign climates, exposed to various causes inimical to physical health, and have often grown grey-haired in the public service. These constitute very different considerations, and at once account for the large mortality invariably prevalent amongst Greenwich and Chelsea pensioners, notwithstanding that individual instances of great longevity are met with at both these establishments.

Another interesting feature characterised the public health of London; namely, the advanced ages attained by many individuals prior to decease. Thus, the deaths of 1,967 persons were recorded who had become 80 years old or upwards, or nearly one in every 30 of the total deaths. Of these, 117—comprising 83 females and 34 males—ranged from 90 to 106 at death. Amongst the 83 females, 54 died from 90 to 95; from that age to 98, 21; 4 succumbed in their 99th year; and four lived to 100. Of the 34 males who had attained 90 or more years, 29 died from that age to 95; at 96 there were two cases; one at 97; another at 99, while only one was reported a centenarian. The oldest person who died during 1856 in London—a female, aged 106—had resided in the Marylebone workhouse during the last 34 years; her son was then alive at the age of 84; which fact appears not only strong proof of her own very protracted age, but likewise an illustration of longevity being hereditary in her family.

It becomes an important inquiry to ascertain if long life be equally frequent in the French metropolis, especially as it constitutes the next most populous city of Europe, and therefore forms a very good basis for comparison. As it is not yet possible to specify the ages of those who died in Paris during 1856, reference must be made to 1855, when the deaths of only 15 persons—5 men and 10 women—whose ages ranged from 95 to 99 were recorded, none being a centenarian. However, to prove conclusively that very old age is not characteristic of Parisians, some curious data, collected during a visit paid to Paris last autumn, may be adduced. In the Bicêtre, which, besides containing about 1,000 male lunatics, is the great poor-house for the Seine department, and mustered 2,794 residents when I visited it in September 1856, the oldest living person was 97, while five were from 90 to 92 years of age; but I could hear nothing of any centenarian. At the Salpêtrière, where all indigent aged females of Paris, besides the pauper lunatics—about 1,200—are taken care of, and which contained 4,237 residents at the period of my inspection, there were only three inmates who had passed their 90th year, the most aged being then 93. This fact is conclusive, and shows the rarity of extreme ages amongst Parisian females, compared with the same class in similar London establishments, where several centenarians are now living. But still more convincing data, supporting such conclusion, may be mentioned. During the last twenty-five years, only one person residing at the Salpétrière has been classed as a centenarian; this occurred fourteen years ago, when a female died at the age of 104, according to the register of that institution. Again, at the Hôtel des Invalides, where the aggregate population usually exceeds 5,000 persons, the most aged veteran on the books was only 91, when the inquiry was made: whereas, at Chelsea hospital—the analogous public establishment in London—the oldest living individual now resident therein has attained his 103rd year. Besides these illustrations of the extreme terms of human life met with, it may also be added that, from all the inquiries made when in Paris, the oldest person actually known to any of my acquaintances was a gentleman reputed then to be 94 years old; and regarding the accuracy of which, little doubt existed in the mind of my informant.

Lastly, as additional evidence showing that fewer examples of very old people are met with in Paris than in London, attention should be directed to the ages recorded on the tombstones erected in its cemeteries. These were examined during my peregrinations last autumn; when few exhibited such high figures as are commonly seen on the banks of the Thames. In Père la Chaise, a large proportion of the death memorials indicated, by the figures inscribed, that the parties buried were by no means old, numbers being very young; several, indeed, ranged above 80, and some even 95 or upwards, but none were centenarians. At Mont Parnasse cemetery—also a very large burying-ground—the highest age noticed was 96, a female; one very remarkable exception, however, deserves especial mention. The monument now alluded to is in the upper or south alley, and bears this inscription,—"Eliza Béens, died December 1854, *in her* 100*th year*, beloved and respected by all who knew her." The words now copied, and their national characteristics, show unmistakably whence she came originally. Consequently, the only reputed centenarian, regarding whom, through recent grave-yard researches in Paris, authentic information could be obtained, was not a native of the country where she had been interred, but in all probability, relying on the brief narrative just quoted, was an immigrant from the British Isles.

Amongst numerous very advanced ages often recorded in London cemeteries, the highest figures actually seen by me on any metropolitan gravestone, occur in the burying-ground of Christ Church, Broadway, Westminster. A square slab, fixed in the eastern wall of that enclosure, states, "Near this place lieth Margaret Patten, who died June 26th, 1739, in the parish workhouse, aged 136 years." Two other centenarians are also buried within the same precinct; one being a female, the other a male, named Man, who was 102 at death. But the oldest person believed to have ever lived in modern London, according to a list which I have collected of 208 reputed metropolitan centenarians—122 being females, and 86 males—was Thomas Carn, who is reported, in the Parish Register of St. Leonard's, Shoreditch, to have died on January 28th, 1588, at the marvellous age of 207 years! he having been born A.D. 1381, during Richard the Second's reign. If this statement be credible, Mr. Carn becomes the most remarkable instance of great longevity yet known in English history.

Reviewing the aggregate facts previously detailed, and every collateral circumstance, impartial observers are fully justified in considering that the French metropolis is neither so salubrious, nor contains the same proportion of patriarchal inhabitants as the capital of Great Britain, notwithstanding its enormous and over-crowded population, and although it is exposed to powerful artificial causes inimical to health. Speaking generally, while in London 22 of every 1000 persons annually fall victims of disease, upwards of 34 individuals are similarly cut off in Paris; or for every two deaths in the former city, three are recorded in the latter, if calculated according to their respective populations; while a smaller number attain very protracted terms of life, and more die in early youth. This latter observation is proved by the fact that the proportion of deaths in Paris, among young persons under five years of age, usually ranges from 48 to 49 per cent. of the total mortality; whereas the analogous ratio last year in London was only 40. On the other hand, in Glasgow, the ratio was 52.9 per cent.; and in Dundee above 55; thereby giving an excess of 15 per cent. in the latter town over the English metropolis. This becomes an important feature, when compared with the capitals of France and England, and shows that some great social evils must prevail in the two northern localities above designated, which need investigation and speedy amendment. Besides the characteristics which have just been mentioned respecting the British metropolis, a larger amount of persons, as previously stated, are there recognised who have lived beyond the ordinary term of human existence, than are found in any other populous district throughout Europe.

As additional evidence deserving record, and as the result of observations made during an extensive holiday tour recently made through Northern Germany, Sweden, and Denmark, I would mention, in reference to the longevity commonly characterising natives of these countries, that none of the lunatic asylums, workhouses, prisons, or other public institutions I then visited—and they were numerous—possessed residents who were actually 90 years old. In no monument in the churches or cemeteries, purposely inspected, did the age of any person so recorded exceed that period; while examples of centenarians were said to be exceedingly rare throughout. The only instance anywhere reported of that kind, is a woman now alive in a village about twenty miles from Stockholm, who, my informant said, had attained 101 years. Further, the oldest living individual I heard of in Denmark, was the mother of a distinguished medical practitioner of Copenhagen: she, as he told me in the course of conversation upon this subject, had then seen 95 summers, and enjoyed good bodily health.

---

